# Fatal intoxications and inherited cardiac disorders in the young: where to draw the line?

**DOI:** 10.1007/s00414-025-03439-9

**Published:** 2025-02-12

**Authors:** Simone Grassi, Fabio Vaiano, Alexandra Dimitrova, Chiara Vullo, Emma Beatrice Croce, Riccardo Rossi, Vincenzo Arena, Sabina Strano Rossi, Oscar Campuzano, Ramon Brugada, Antonio Oliva

**Affiliations:** 1 Forensic Medical Sciences, Department of Health Science, University of Florence, Florence, Italy; 2 FT-LAB Forensic Toxicology Laboratory, Department of Health Science, University of Florence, Florence, Italy; 3 Section of Legal Medicine, Department of Health Surveillance and Bioethics, Fondazione Policlinico A. Gemelli IRCCS, Università Cattolica del Sacro Cuore, Rome, Italy; 4 Area of Pathology, Department of Woman and Child Health and Public Health, Fondazione Policlinico Universitario A. Gemelli IRCCS, Rome, Italy; 5 Medical Science Department, School of Medicine, Institut d’Investigacions Biomèdiques de Girona (IDIBGI), University of Girona - Cardiovascular Genetics Center, University of Girona, Girona, Spain; 6 Centro de Investigación Biomédica en Red en Enfermedades Cardiovasculares, Madrid, Spain

**Keywords:** Sudden cardiac death, Sudden death, Cardiomyopathies, Channelopathies, Drugs

## Abstract

Sudden cardiac death represents a significant public health concern and is one of the leading causes of early mortality worldwide. The escalating use of illicit drugs, approximately 269 million people in 2018, represents a growing public health. Some of these drugs are stimulants that may have multiple effects on the cardiovascular system including the cardiac rhythm, then substance abuse increases the risk of sudden death. For instance, drugs like cocaine and methamphetamine, may be responsible for myocardial infarction as well as occlusive coronary thrombosis with acute infarction. The consequences of such occurrences are far-reaching, with considerable effects not only on the victims but also on their families. Sudden cardiac death presents considerable forensic diagnostic challenges, particularly in the presence of high but non-lethal drug levels increasing the possibility of a genetic predisposition to malignant arrhythmogenic events. Our review aims to discuss the complex relationship between illicit drugs and congenital cardiac disorders, stressing the forensic issues deriving from their interaction and from the differential diagnosis. Indeed, especially when a non-lethal dose of illicit drug in presence of ambiguous microscopic findings is reported, being able to discriminate between a toxic sudden death (entailing criminal implications for the drug dealer) and a natural sudden death is a forensic issue of upmost importance.

## Introduction

Sudden death (SD) is defined as the decease of a healthy individual within an hour from the first symptoms or, if the lethal episode was witnessed, within a day since the victim was last seen in good health. When it has a cardiac cause, it is called sudden cardiac death (SCD), a condition mainly due to congenital cardiac disorders (i.e., inherited arrhythmogenic syndromes -IAS-) in those younger than 35 years [1, 2]. Globally, the incidence of SCD among young individuals ranges from 0.8 to 6.2 per 100,000 persons annually. The main forensic challenge associated with SCD in the young is the significant prevalence (approximately 30%) of cases where a definitive cause of death cannot be determined through autopsy. Most of these cases are attributed to cardiac channelopathies (which generally relate to inconclusive autopsies) or cardiomyopathies (which can cause fatal electrical events even when the macroscopic phenotype is mild or absent) [2]. Illicit drugs also are a very frequent cause of SD and SCD (especially in the 15–34 age group), even after a single assumption [3–5]. Indeed, in most of the cases of SD of those younger than 50 years, post-mortem toxicological testing is positive. Positive toxicological testing is defined as the presence of illicit substances (i.e., cocaine, cannabis, amphetamines or polysubstance abuse) [6, 7].

In particular, there is a strong correlation between SCD and illicit drugs assumption: for instance, prevalence of cocaine acute intoxication is 13–58 times higher in SCD victims than in general population [8]. Toxic SCD are generally thought not to correlate to specific macroscopic or microscopic features, an issue that is shared with all the cases of cardiac channelopathies and many cases of cardiomyopathies (especially in paediatric age), that can show subtle, mild or ambiguous signs at the autopsy [9]. This problem is extremely significant because– as said– SCDs due to intoxications and congenital cardiac disorders mainly insist in the same age group, despite having radically different legal and public health implications. Indeed, congenital cardiac disorders are frequently inherited following an autosomal dominant pattern, have incomplete penetrance, variable expressivity, and (especially cardiac channelopathies) tend to have SD as first clinical manifestation usually caused by a trigger, such as drugs. Thus, they represent a public health issue, being the forensic diagnosis of fundamental importance for early diagnosis and management of these conditions in the first-degree family of the victims. On the other side, toxic SDs have clear legal implications (i.e., in many countries, like Italy, the dealer can be prosecuted for murder), also when the substance is “only” the trigger of a preexisting congenital condition. Hence, in this narrative review we aim to describe the microscopic and macroscopic features recurring in fatal intoxications, in order to underline how they can be differentiated from “natural” cardiac causes of SD.

## Methods

For this review, a literature search was performed through various resources (i.e., PubMed, ResearchGate, Embase) from 1974 to 2024. The search terms included: sudden death, sudden cardiac death incidence, cardiomyopathies, channelopathies, illicit drugs, toxicology, molecular autopsy. The research question covered particularly what microscopic features recur in deaths caused by main substances of abuse (alcohol, cocaine, amphetamines, cathinones, opioids, cannabis, and hallucinogens). Therefore, papers were eligible if the correlated intoxication caused by one of the aforementioned substances to clinical/pathological signs able to explain/suggest the cause of the sudden death. There were no restrictive inclusion criteria regarding the type of paper (original research papers, reviews and case reports were considered). Papers not written in English were excluded. Despite the wide time span considered for the search (chosen not to exclude valuable source of information), papers with modern methodological approach for toxicological (gas chromatography–mass spectrometry and liquid chromatography–mass spectrometry) and genetic (whole genome, whole exome or targeted next-generation sequencing) analysis were preferred. Since the broadness of the research question, a non-systematic (narrative) approach was chosen for the review. Two independent authors performed the review, eliminating the duplicates after searching on different databases and comparing the findings, with full concordance of the results.

## Results

Results are summarized in Table 1, specifying for each illicit drug the main cardiovascular pathological findings and (if available) the blood concentrations for clinically evident toxic effects and coma/deaths.

**Table 1:** Association between illicit drug, main recurring microscopic features and toxic/fatal concentrations

Drug	Main cardiovascular pathological findings	Clinically evident toxic effects blood concentrations (mg/L) [10,11]	Coma/Death blood concentrations (mg/L) [10–12]
Cocaine	Cardiomegaly, hypertrophic/ dilated cardiomyopathy, acute myocardial ischemia/ infarction (with occlusive coronary thrombosis), myocarditis with necrosis and fibrosis, myocardial contraction bands, vasculitis [13–19]	0,25/0,50	0,9
Amphetamines	Acute myocardial infarction; arterial dissections; dilated/ hypertrophic cardiomyopathy; myocarditis with necrosis and fibrosis; increased perivascular and interstitial fibrosis [4,16,20–24]	0,2 (methamphetamine) 0,2 (amphetamine) 0,35 (3,4-Methylenedioxymethylamphetamine– ecstasy)	1 (methamphetamine) 0,5 (amphetamine) 0,4 (3,4-Methylenedioxymethylamphetamine– ecstasy)
Cathinones	Myocardial infarction; dilated cardiomyopathies; myocarditis [25,26]	0,001–8,400 (MDPV) 0,001 − 0,606 (α-PVP)	0,001–29 (MDPV) 0,001–20 (α-PVP)
Opioids	No common microscopic features	0,1 (morphine) 0,3/0,6 (methadone)	0,1 (morphine) 0,4 (0,05 − 1,00) (methadone)
Cannabis	Myocardial ischemia/ infarction; thrombi; cardiomyopathies (like Takotsubo, with contraction band necrosis); arteritis [4,27–34]	undetermined	undetermined
Hallucinogens	myocardial infarction; Takotsubo cardiomyopathy [35–38]	0,001 (LSD) 0,018 (psilocin)	0,002 − 0,005 (LSD)

### Alcohol

Acute alcohol intoxication, especially when the consumption is heavy, has a negative inotropic effect, potentially leading to atrial arrhythmias (like atrial fibrillation) [39]. One of the long-term effects of alcohol abuse is the so-called alcoholic cardiomyopathy, characterized by dilatation and potential wall thinning of the left ventricle, associated to myocardium hypertrophy and replacement fibrosis [39]. The relationship between inflammation and alcohol is particularly complex, because low doses of alcohol are associated to reduced inflammation, while a long-term heavy consumption implies an oxidative stress, leading– among the others - to an accumulation of acetaldehyde, a decrease in antioxidant enzyme levels, and an alteration in neurohormonal systems [39].

Forensic cases of SCD with acute alcohol intoxication cover a wide age range, but, differently from illicit drugs, mainly regard non-young individuals [40–42]. Forensic studies tend to suggest a possible role of alcohol in SCD: for instance, Holmström et al. found that more of a fourth of forensic cases of SCD had alcohol in blood and/or urine, with most of them (86%) showing higher levels in urine than in blood (suggesting a late stage of inebriation) [40]. Moreover, they compared autopsy findings in those during the late stage of inebriation with those died during the early stage, observing in the first group higher prevalence of fatty liver and lower prevalence of myocardial fibrosis and clinical diagnosis of cardiovascular disease [40]. Perkiömäki et al. also evaluated forensic cases of SCD, finding that the 38% of them had a blood ethanol concentration above 0‰ [41]. Among the positive cases, the 41% showed a concentration ≥ 1.5‰ and the 56% ≥1‰ [41]. Regarding autopsy findings, among SCD victims with alcohol in blood, Kauppila et al. found relatively high prevalences of cardiomyopathies (in particular, alcoholic cardiomyopathy and hypertensive cardiomyopathy– i.e., left ventricular hypertrophy with unspecific fibrosis of the myocardium, arterial medial hypertrophy and intimal fibrosis in renal arterioles) and myocarditis [42]. A specific issue is represented by the so-called binge drinking (heavy alcohol consumption over a short period of time), that has been suspected to have a pro-arrhythmic effect since the 1978 Ettinger et al.’s paper [43]. In 2022, Tu et al. found that alcohol consumption was not a risk factor for ventricular arrhythmias but still had a U-shaped association with SCD, with intake lower than 208 g per week showing the lowest risk, while greater consumption of specific beverages (beer, spirits, and cider) related to an increase in SCD risk [44]. On the other end, in 2024 Park et al. studied a population of 397.164 individuals, finding that alcohol intake was neither a protective factor nor a risk factor for SCD, and this result was valid even for the carriers of variants of genes involved into alcohol metabolism [45].

### Cocaine

**Fig. 1:**
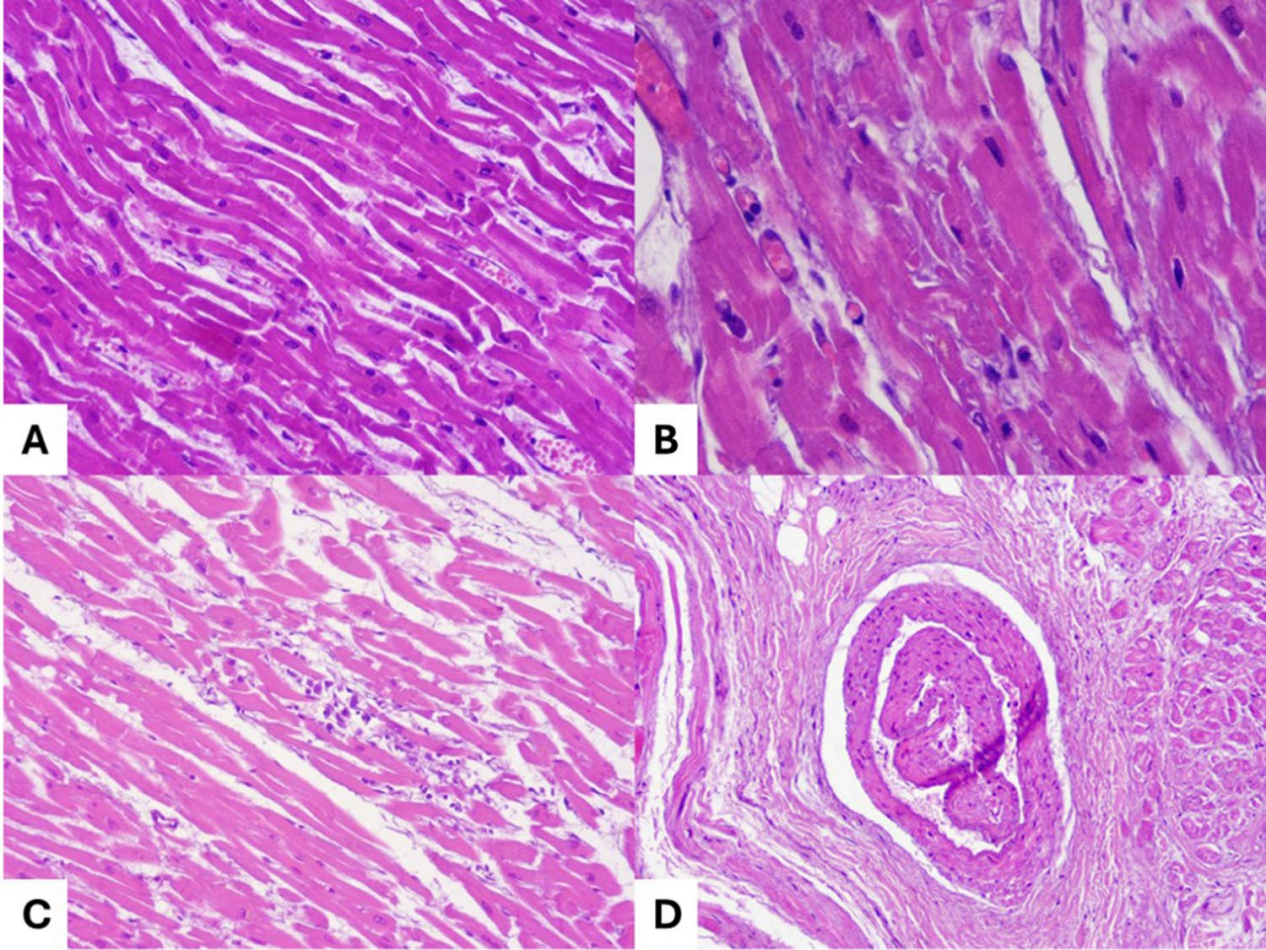
Left ventricular (**A**, **B**, **C**) and atrial (**D**) examination in a chronic abuser of cocaine who died in a state of acute cocaine intoxication (blood concentration in the femoral artery: 315 ng/mL): myocardial wavening (**A**); foci of coagulative necrosis (**B**); areas of myocarditis (**C**); arterial dissection in the area next to the sino-atrial node (**D**)

Cocaine use can cause electrical anomalies, like Brugada Syndrome (BrS) and QT prolongation and consequent *torsade de pointes* - in cases of both acute intoxication (due to increased excitability of the heart) and chronic intoxication (due to myocardial fibrosis) [13]. In acute intoxication, pro-arrhythmic effects of cocaine are enhanced by the (frequent) combination with alcohol, because cocaethylene (a metabolite of these substances) inhibits inward rectifying potassium channels [14]. Regarding structural changes, cardiomegaly can be found in up to one third of the cases [15]. Moreover, cocaine stimulates coronary vasoconstriction, stimulates chronotropic and inotropic drive, then causing ischemic injury to the myocardium [13]. Indeed, myocardial infarction is a very frequent finding in cocaine intoxications: abusers are exposed to a high risk of infarction in comparison with general population (odds ratio up to 6,9) and this risk is particularly high (increased by 24-hold) in the first hour of acute [16]. There is not a clear dose-dependent risk of myocardial infarction in cocaine abusers, since in these cases even serum concentrations as little as 0,1 mg/L have been reported [3]. Moreover, most of the cocaine-related myocardial infarctions occurs in occasional users rather than in frequent users [3]. When associated to cocaine intoxication, myocardial infarction can be both acute with occlusive coronary thrombosis and remote with organized, recanalized thrombus [3]. However, SCD related to left ventricle dysfunction in cocaine acute intoxication can also occur without any sign of ischemic injury due to the potential negative inotropic and lusitropic effects of high dose of the substance [13]. Left ventricle failure caused by toxic dilated cardiomyopathy (DCM) is a recurring feature and it poses the issue of differentiating it from other (e.g., congenital) form of DCM. Indeed, cocaine-related DCM does not show specific features, having been solely associated to increased myocardiocytes’ volumes and possible presence (up to 20% of cases) of myocarditis (e.g., eosinophilic myocarditis) [17]. In general, subendocardial ischemia, myocarditis with necrosis, fibrosis of the myocardium and left ventricular hypertrophy are recurrent findings in these cases (Fig. 1) [16, 18]. Moreover, at post-mortem investigation myocardial contraction bands (like those of Takotsubo syndrome) may be found, due to the cathecolamine-driven stress [19]. At the autopsy, in chronic abusers (especially if they also smoked cigarettes) thrombi (generally associated to advanced atherosclerosis) should also be carefully searched, since the prothrombotic effects of the substance [13]. At the same time, signs of vasculitis both in the nasal cavities and at systemic level should be searched, since it can be caused by both cocaine and its adulterants (e.g., levamisole) [13]. In general, accurate analysis of the main arteries is crucial at the autopsy: cocaine can cause vascular smooth muscle cell apoptosis and cystic medial necrosis, potentially leading to dissection of aorta, coronary or carotid [13]. It should be considered that these latter findings are likelier in crack cocaine smokers, since this drug is usually used in short intervals due its duration of action [13].

### Amphetamines

Amphetamines can cause myocardial injury both directly (e.g., increased oxidative stress, promoted apoptosis, mitochondrial dysfunction, alteration of gene expression) and indirectly (e.g., vasospasm of epicardial coronary arteries) [20]. Indeed, in the young population, their use has been significantly associated with acute myocardial infarction [21]. Methamphetamines can also cause reversible remodelling of cardiac ion channels, leading to arrhythmias [16]. Stimulants intoxication should be carefully differentiated from Long QT syndrome (LQTS), since they can prolong QT causing the same kind of arrhythmias of this condition (*torsade de pointes*) [22]. Even in healthy young cases, relevant post-mortem features can be found at both macroscopic (e.g., cardiomyopathies– especially with ventricular dilatation, possible even in those who have used the substance for less than 6 months) and microscopic level (e.g., myocardial fibrosis, myocarditis with cellular vacuolization, hypertrophy, focal necrosis) [16, 20].

In particular, autopsy studies showed that abusers of methamphetamines frequently present significant perivascular and interstitial fibrosis in the myocardium, especially in the left ventricle [23]. Fibrosis, together with ventricular hypertrophy and increased heart weight, has been related to the typical “binge and crash” (very intense assumption of the stimulants followed by a period of withdrawal) pattern of assumption in animal models [23]. Other kinds of injuries caused by amphetamines are valvular heart diseases, thrombosis and vasculitis (through proinflammatory immunoactive glycation end products) [4, 22]. Vasospasm is a critical effect of these substances, since it can cause vessels’ rupture– leading, for instance, to intracranial haemorrhages [24].

Finally, there is currently no evidence linking therapeutic use of amphetamines (mainly for ADHD, clinical studies are still in the trial phase) and sudden cardiac death in the young, while this association– still controversial - has been supported in adult subjects [22, 46, 47].

### Cathinones

Cathinones have been associated to many potentially fatal cardiac events, like cardiac arrest, tachyarrhythmias, myocardial infarction, DCM and myocarditis [25]. Despite their cardiotoxicity has been clearly described yet, being structural analogues of β-ketone amphetamines, they could affect similar to amphetamines [26].

### Opioids

Opioids’ cardiac effects largely depend on the specific substance: for instance, methadone, oliceridine, l- α-acetylmethadol, and fentanyl may prolong QT blocking inward potassium rectifier channels, while some fentanyl derivates cause bradyarrhythmias [48, 49]. In particular, QT prolongation is a common finding in methadone users, correlating to dose- and time-in-treatment, while it is rarer in chronic heroin abusers (34% vs. 19%) [27]. Accurate sampling and testing for toxicological purposes is of utter importance in arrhythmic deaths to weight the potential role of the opioid, since there is a well-known association between risk of fatal arrhythmias and different doses of specific substances– like methadone [50]. Like in many cases of arrhythmic deaths, SCD due to opioids generally are autopsy negative [50]. Another challenge is represented by the fact that opioids are generally injected, and thus cardiac anomalies could be due to infectious diseases transmitted through infected needles (e.g., endocarditis, mainly of the tricuspid valve) [51]. No significant microscopic changes have been related to opioids’ use, albeit Greenwald et al. reported that past-month number of days of heroin use predicted lower likelihood of left ventricular hypertrophy [27]. An issue that is common among all the drug users but is particularly frequent in opioids’ abusers is the use of other drugs just before/after or together with the opioid [52]. Clinical differentiation with arrhythmogenic syndromes like LQTS can be particularly difficult when the opioid is used in combination with drugs that prolong QT, like cocaine, amphetamines and high-dose benzodiazepines, or when there is a combined use of opioids (like heroin and methadone) [52, 53]. However, Greenwald et al. found that use of heroin and cocaine caused modest incremental variance in QT interval, stressing the contribution of other variables in electrical anomalies, like race, body mass index and use of tobacco, alcohol and/or cannabis [27].

Patients undergoing methadone-based opioid substitution therapy might misuse benzodiazepines, for instance to relieve withdrawal symptoms or to enhance the effects of the opioids [54]. Indeed, Errico et al. reported that this combination accounted for 27.6% of deaths associated with methadone use. Histological findings highlighted myocardial hypertrophy and cardiomegaly in 19.2% of cases, while contraction band necrosis and coagulative necrosis were identified in 11.5% of cases. The authors observed that the risk of central respiratory depression, cardiovascular failure and then death is particularly high in individuals who are not undergoing supervised methadone therapy, underscoring the need for careful monitoring and management of such treatments [54]. Finally, cardiac causes of death could be due to their adulterants, like clenbuterol– that can cause direct myocardial injury [52].

### Cannabis

Cannabis use exposes both acute and chronic users to acute coronary syndromes-transient myocardial ischemia (mainly to induced coronary vasospasm), thrombotic events-peripheral arteriopathies and pro-arrhythmic effects [4, 28–33]. Moreover, both natural and synthetic cannabinoids had been associated with cases of myocardial infarctions in healthy young subjects [4]. The effect of cannabis on heart rate largely depends on dose: at low-moderate doses it causes tachycardia, while at high doses bradycardia [4]. Moreover, daily cannabis use has been reported as a predictor of shorter QT interval [27]. Some authors reported that cannabis effects may evocate a characteristic ST-segment elevation in the electrocardiogram (ECG) usually related to BrS, inhibiting sodium and potassium currents [34, 55]. Cannabis use has also been related to cardiomyopathies, in particular Takotsubo cardiomyopathy– a condition characterized by ballooning of the ventricles and contraction bands necrosis of the myocardium [33]. Finally, some authors found recurrent cases of arteritis in those exposed to high doses of cannabinoids [33]. However, in most of cannabis-related deaths there were preexisting disorders of medical interest, like arrhythmogenic cardiac condition [56, 57].

### Hallucinogens

Many hallucinogens have been associated with arrhythmogenic phenomena. For instance, ingestion of ibogaine has been reported to cause QT prolongation and consequent torsade de pointes [58, 59]. “Magic mushrooms” with psychotropic effects largely caused by psilocybin and psilocin compounds, have also shown cardiotoxic effects in reported cases, including prolonged QT intervals, ST-segment elevation, myocardial infarction and Takotsubo cardiomyopathy [35–38]. LSD intoxication include cardiovascular alterations as sinus tachycardia and subsequent myocardial infarction [60]. Moreover, LSD has also been associated with other “structural” alterations like ischemia caused by lower limb arterial vasospasm requiring percutaneous angioplasty and carotid artery occlusion [60–62]. There have not been recorded deaths due to ayahuasca, but a fatal case of concomitant use of pure 5-methoxy-DMT has been reported [63].

## Discussion

**Fig. 2:**
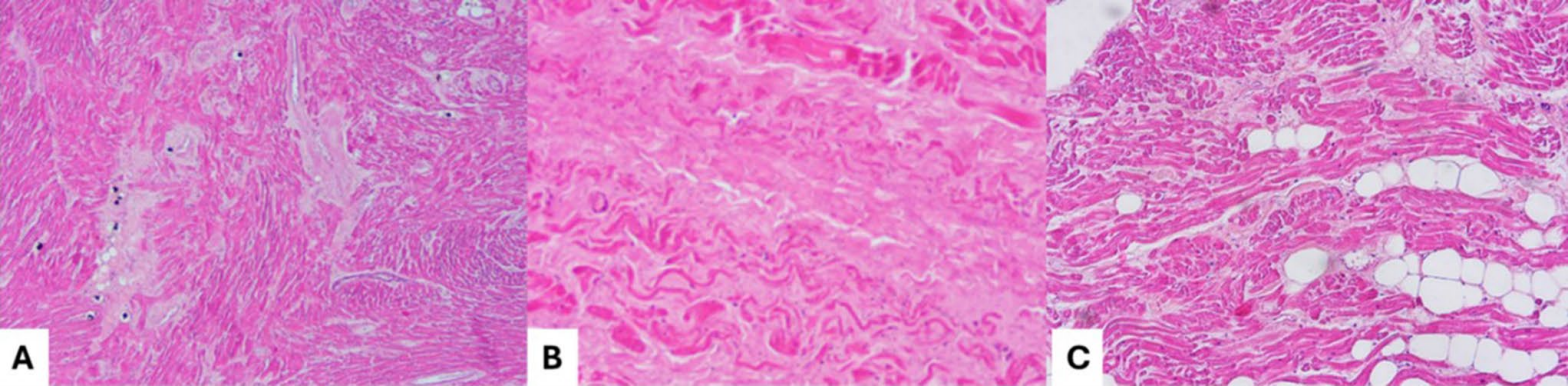
Adapted from [31] the sudden death of a 20-year-old healthy man with recent use of cannabis with multiple cardiopathological anomalies: wavering of myocardial fibers, fibrosis and disarray in the left ventricle myocardium (**A**); fibrosis of the sino-atrial node (**B**); adipositas cordis in the antero-lateral region of right ventricle free wall (**C**)

From a forensic point of view, in SD of young healthy victims, fatal intoxication is one of the first hypotheses to be verified, followed at the autopsy by structural changes, that– as said– can be subtle or ambiguous (like the presence of only disarray and fibrosis in a young victim with genotype positive for hypertrophic cardiomyopathy) (Fig. 2) [64].

Post-mortem genetic testing (also called molecular autopsy) remains a (pivotal) tool for a diagnosis per esclusionem, i.e. when the other hypotheses have been excluded [64]. Among the substances analysed, cocaine emerged as the most extensively studied in relation to SCD [8]. This predominance reflects both the widespread use of cocaine and its well-documented impact on the cardiovascular system. On the other hand, significantly fewer data are currently available regarding the effects of synthetic cathinones, for instance.

As shown in Table 1, common drugs can cause the same microscopic patterns found in congenital cardiac causes of SD, like left ventricular hypertrophy and myocarditis with fibrosis, none of which can be considered specific or pathognomonic. In cases where only these findings are found, in absence of anomalies able to explain death, drug levels in proper matrixes should be carefully considered. Indeed, the mere toxicological positivity of a sample cannot abruptly interrupt forensic investigation if the level is not sufficient to explain death and, in case of inconclusive autopsy, post-mortem genetic testing should be indicated even when toxicological testing is positive.

**Fig. 3:**
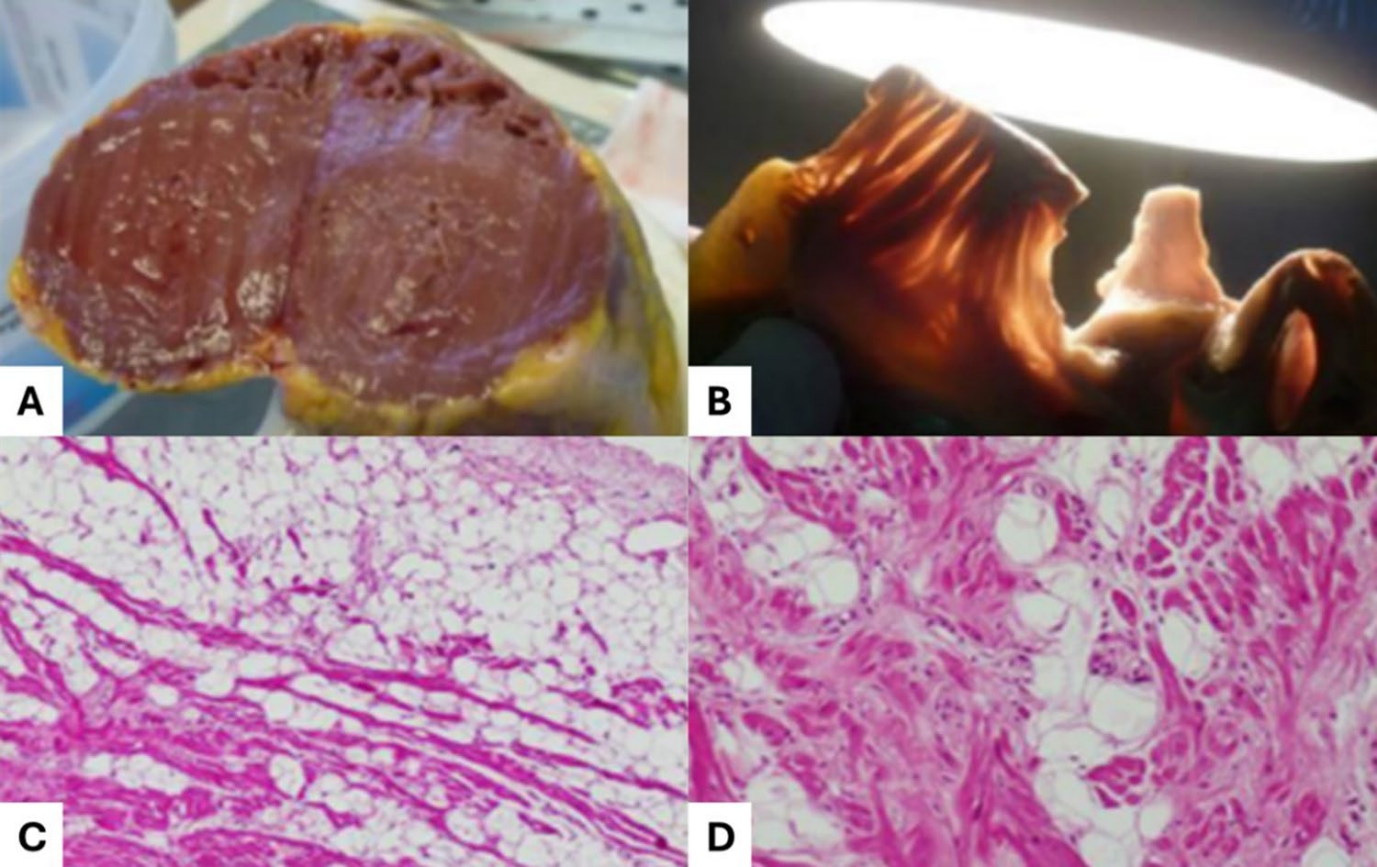
Adapted from [65]: sudden death of a 41-year-old chronic abuser of alcohol and cocaine affected by arrhythmogenic cardiomyopathy: biventricular hypertrophy (**A**); thinning to the apex of the right ventricle at the transillumination test (**B**); fatty replacement of the right ventricle myocardium with fibrosis and some residual myocyte with perinuclear vacuolization and myofibril loss (**C**); right ventricular myocardium fibrosis with initial myocytes trapping (**D**)

In these cases, it can be difficult– but of upmost forensic importance– to verify whether a drug level higher than the toxic concentration but lower than the lethal concentration could have interacted with an abnormal substrate due to a cardiac structural or channel disorder, and for these purposes post-mortem genetic testing is fundamental (Fig. 3).

The importance of this procedure is that in some countries, like Italy, if the drug is demonstrated to have triggered or favoured the outbreak of a latent condition, the drug dealer can be prosecuted for murder.

Throughout investigation is also crucial when the intoxication is due to substances– like cathinones– that are difficult to be tested and whose toxic and lethal levels are often misknown or unknown [12, 66–68].

Moreover, pathologists should always consider that, when the drug level is interpreted, many confounding variables should be taken into account: (i) if the victim was fragile, for instance severely comorbid, a lower dose may be fatal; (ii) if the victim was a chronic user, the fatal dose is usually higher than a naïve user; (iii) factors that may underestimate/overestimate the actual drug level like time interval between death and sampling, sampling choices (e.g., peripheral vs. central blood), use of correct storage procedures (e.g., high conservation temperatures, failure to use preservatives) and analytical procedures [66–68].

Moreover, as shown by our review, there is no strict correlation between drug level and likelihood of toxic SD, being impossible to draw a dose-dependent likelihood distribution of SD caused by drug rather than by other causes.

Accurate clinical information is fundamental for the pathologist, but are often of scarce importance for this differential diagnosis, since– as said - cocaine and cannabis can evocate a characteristic Brugada pattern in the ECG, as cocaine, amphetamines and some opioids can prolong QT [69, 70]. On the other side, history of drug abuse is still frequently underreported, as conditions unrelated to drugs or cardiac disorders that can still explain arrhythmic anomalies [27, 32].

Solid forensic procedure is therefore fundamental. As first step, toxicological testing must be performed in order to marginalize the risk of false positives and negatives: for these reasons, it is usually recommended to perform screening tests at least through immunoassay. Anyway, the optimal approach would be the routine use of chromatographic/mass spectrometric techniques, that should always be used to confirm immunoassay results, due to their capability to detect also new psychoactive substances [71].

Then, accurate cardiac examination must be performed [72]. Vascular anomalies, like intracardiac or extracardiac dissections,– as said– are more frequent in (some) toxic deaths rather than in congenital cardiac disorders. Since the (few) recurring anomalies are almost shared by fatal intoxications and congenital cardiac disorders, attention should also be paid to the appearance and the localization of the anomalies. For instance, asymmetric and significant hypertrophy of the interventricular septum, possibly associated to apical hypertrophy, suggest a congenital condition rather than an acquired cause [73]. In a young case, disarray can be normal in some parts of the heart (typically, the anterior and posterior parts of the right ventricle next to the septum) and thus a “normal” localization should imply supplementary caution in the cardiac exploration [74]. Moreover, localized myocarditis with possible fibrosis in the outflow tract of the right ventricle should entail the hypothesis of a BrS [4, 75].

Herein, we propose this protocol:

In a case of sudden unexplained death, toxicological analysis and traditional autopsy are mandatory. Gross examination of the heart may reveal or not macroscopic anomalies.

Scenario 1: heart with no macroscopic anomalies.

- if there are relevant histologic alterations, it should be assessed if they qualify as cause of the death and it is still important to evaluate if there is evidence of toxicological intoxication and, in this latter case, if the drug levels are lethal - i.e., able to justify the cause of the death. In cases with significant microscopic and toxicological results, the forensic pathologist should carefully evaluate and discuss the hypothesis of an interaction between structural anomalies and intoxication.
- If there are not relevant histologic alterations and there is no clear evidence of a lethal intoxication (e.g., the toxicological testing is negative, the drug level is not higher than the lethal concentration), the forensic pathologist should carefully analyse other sources of information (e.g., familiar anamnesis) to evaluate if a molecular autopsy can be indicated. If the forensic toxicologist cannot exclude an underestimation of the drug levels, a new analysis should be considered before molecular autopsy, if proper matrix is still available.

Scenario 2: heart with macroscopic anomalies.

- if there is evidence of a lethal intoxication, before certifying the cause of the death, the forensic pathologist should still revise all the available information, including familiar anamnesis, to verify if a molecular cause of sudden death can confidently be ruled out without post-mortem genetic testing (that remains a tool for per-esclusionem diagnosis).
- if the drug levels are not lethal, the forensic pathologist should verify if the alterations can reliably be considered the cause of the death or, if the cause of the death is still uncertain, indicate a molecular autopsy.

**Fig. 4:**
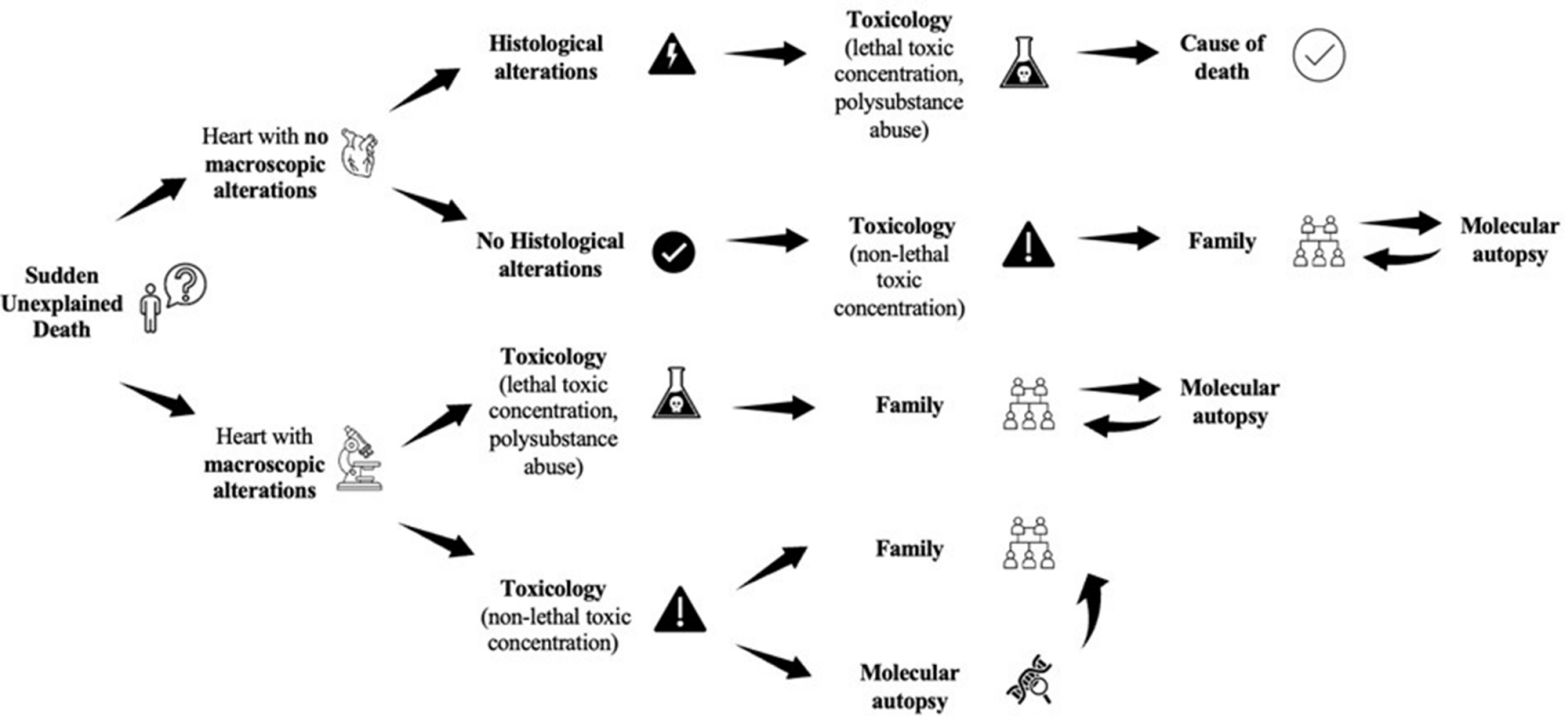
Workflow diagram for two autopsy scenarios involving the use of illicit drugs. In cases negative at the autopsy (no macroscopic alteration), with a normal heart, toxicological information should always be carefully considered, with molecular autopsy potentially being indicated if the identified drug concentration is not lethal - low or even negative. If anomalies are found at the cardiac exploration, even if the toxicological values are lethal, a molecular autopsy may still be considered because of the possibility of an interaction between drug and congenital abnormal substrate

This workflow is summarized in Fig. 4.

## Conclusion

A sudden death event, especially in the young population, suggest a comprehensive medio-legal autopsy. In cases without a conclusive cause of the unexpected decease, in particular when toxicological testing is negative or does not show lethal concentrations, molecular autopsy should always be considered. A multidisciplinary analysis of the data, combining the toxicological, pathological and genetical perspectives is recommended to unravel the origin of the lethal event.

## Data Availability

The datasets generated during and/or analysed during the current review are available from the corresponding author on reasonable request.
